# Personality and mentalization: A latent profile analysis of mentalizing problematics in adult patients

**DOI:** 10.1002/jclp.23430

**Published:** 2022-08-17

**Authors:** Giulia Gagliardini, Salvatore Gullo, Arianna Teti, Antonello Colli

**Affiliations:** ^1^ Department of Humanities “Carlo Bo” University of Urbino Urbino Italy; ^2^ Department of Psychology, Educational Science and Human Movement University of Palermo Italy Palermo Italy

**Keywords:** assessment, mentalization, personality disorders, reflective function

## Abstract

**Background:**

The aim of this study was to investigate the relationship between patients' mentalizing problematics and their personality; specifically, it aimed to identify clusters of individuals characterized by specific patterns of mentalizing imbalances and to analyze the relationship between these and diagnosis of personality disorder (PD), nonmentalizing modes, emotion dysregulation, and interpersonal reactivity.

**Methods:**

Four hundred therapeutic dyads were recruited. A part of these (*n* = 183) only completed clinician‐report measures, Mentalization Imbalances Scale, and Modes of Mentalization Scale, while others (*n* = 217) also completed patients' self‐report measures, which were Reflective Functioning Questionnaire, Difficulties in Emotion Regulation Scale, and Interpersonal Reactivity Index.

**Results:**

A latent profile analysis enlightened the presence of four clusters with problematics in the dimensions of mentalization, indicated by cluster names: (1) Affective‐self‐automatic profile (ASA‐P) (with higher percentage of PDs); (2) External profile (E‐P) (with lower percentage of PDs); (3) Others‐automatic‐affective profile (OAA‐P); (4) Cognitive‐self‐automatic profile (CSA‐P). Multivariate analysis of variances confirmed that the four clusters differed in relation to the quality of mentalization, emotional dysregulation and interpersonal reactivity, with higher levels of nonmentalization modes, uncertainty about mental states and emotion dysregulation in ASA‐P, higher levels of good mentalization in E‐P, lower impulsivity in CSA‐P, and greater empathic concern in OAA‐P.

## 1 INTRODUCTION

Although there are several theoretical and clinical models for the understanding of personality disorders (PDs), there is widespread agreement that individuals need to understand internal mental states to form stable and coherent self‐representations, and that they must understand others' mental states to form and maintain interpersonal relationships (Bateman & Fonagy, 2016, 2019; Dimaggio et al., 2007). In this context different authors have stated that mentalization, which has been defined as “the mental process by which an individual implicitly and explicitly interprets the actions of himself and others as meaningful on the basis of intentional mental states such as personal desires, needs, feelings, beliefs, and reasons” (Bateman & Fonagy, 2004, p. xxi), may have a crucial role in the development of personality pathology.

More in detail, from the perspective of the theory of mentalization, patients with personality pathology may be characterized by the prevalence of nonmentalizing modalities of thought (rather than by a solid mentalization), such as concrete thought or psychic equivalence (patients experience reality and the inner world as a whole, in a sort of isomorphism, in which projections of fantasies into their external world are felt as real), teleological thought (which characterizes those situations in which the physical and observable dimension is dominant, and information on the inner world is gained from the external reality), and pseudomentalization (situations in which patients understand and reflect on mental states but these reflections are not connected with reality: mentalization is an intellectual game and is not related to the real experience) (Bateman & Fonagy, 2016).

Different authors have developed this hypothesis by enlightening how patients with PDs may be characterized by several problematics in mentalization (see e.g., Beeney et al., 2015) and by the presence of nonmentalizing modalities of thought (see e.g., Bateman et al., 2013), and have suggested the hypothesis that each PD may be characterized by specific problematics in mentalization (Semerari et al., 2014). However, this conceptualization of personality pathology requires for us to adopt a multidimensional lens on mentalization, which initially had been operationalized in monodimensional terms by using the Reflective Functioning Scale (RFS; Fonagy et al., 1998). In further development of the definition of the construct, mentalization has been defined as composed of eight dimensions (Fonagy et al., 2012; Luyten et al., 2012): automatic (implicit) mentalization (refers to the implicit and unconscious processes of recognizing and understanding inner mental states in oneself and others), controlled (explicit) mentalization (refers to a conscious and deliberate process), mentalization toward self (refers to the capacity to reflect on our own inner experiences), mentalization toward others (refers to the capacity to understand the underlying reasons for others behaviors correctly), cognitive mentalization (refers to the activity of “thinking about thinking” and of understanding the representational nature of thoughts), affective mentalization, that is a particular type of affective regulation that is composed of three different domains (Jurist, 2005): identifying, processing, and expressing affective mental states; internally focused mentalization (refers to a focus on mental states, e.g., thoughts, feelings, or emotions), and externally based mentalization (refers to mental processes based on actions or physical characteristics). A good capacity to mentalize has been described as the result of an equilibrium between the different dimensions of the construct, while difficulties in mentalization can manifest throughout specific imbalances on one or more facet of the construct (Fonagy et al., 2012).

Different psychopathological domains, especially PDs, can be interpreted as the result of an imbalance on one or more of the dimensions of mentalization (e.g., Bateman et al., 2013; Gagliardini et al., 2018). Narcissistic PD, for example, seems to be characterized by a marked capacity to cognitively mentalize which is, however, linked to a form of detachment from the affective facets of experience (Blair, 2008; Blatt, 2008; Drozek & Unruh, 2020; Luyten et al., 2012). On the contrary, borderline and histrionic PDs seem to be linked to imbalances on affective mentalization (Blatt, 2008; Gagliardini et al., 2018; Lowyck et al., 2016; Semerari et al., 2014) and a lack of certainty about feelings (Beeney et al., 2015) which does not seem to be paired by the capacity to explicitly and cognitively mentalize. Some authors have also hypothesized that patients with dependent, obsessive–compulsive, and avoidant PDs (“Cluster C” disorders, according to the DSM‐5; American Psychiatric Association [APA], 2013), may share problems related to alexithymia, or a difficulty at focusing, identifying, and describing feelings (Moroni et al., 2016) and may therefore be characterized by an imbalance in the cognitive dimension of mentalization. Moreover, dependent PD may be characterized by an imbalance on the others (Bateman & Fonagy, 2016, 2019; Gagliardini et al., 2018), in line with the consideration that among the core features of this disorder are the tendency to excessively rely on others for everyday decisions and the fear of loss and abandonment (APA, 2013).

The aforementioned studies have tried to investigate the relationship between mentalization and personality pathology. However, there are some methodological issues which have to be addressed. Most notably, in the cited studies the authors have adopted a monodimensional approach and have chosen to use the RFS, a monodimensional measure of mentalization widely used in clinical practice but which fails to rate problematics related to the specific mentalizing dimensions; different studies have tried to overcome this limitation by using measures developed to assess different constructs (e.g., emotion regulation or empathy; Beeney et al., 2015), which however can only assess single facets of mentalization and not all of its dimensions. Empathy, in fact, is a multidimensional construct that involves different abilities related to the capacity to identify and understand others' emotions and feelings and perspectives (Albiero et al., 2006), while emotion regulation refers to the capacity to identify, accept, and control our own emotions (Gratz & Roemer, 2004). Both these capacities can be affected by problematics in mentalization. However, although mentalization is a construct in the literature strongly linked and partly overlapping to empathy and emotional regulation (e.g., Hooker et al., 2008), some important differences also exist among these. Empathy, unlike mentalization, is basically associated to the capacity of sharing emotional states with other people, is modulated by immediate sympathetic circuits (Cerniglia et al., 2019). From a theoretical point of view, mentalization and emotional regulation seem more closely associated, and for example, Sharp et al. (2011) argued that good ability in mentalizing guides the individual's emotional response:  In these terms mentalization can be conceptualized as a component of emotion regulation. Fonagy et al. (2002) hypothesized that emotion regulation dysfunctions may obstacle the interpersonal relationships through which mentalizing is normally acquired and thus determine dysfunctions in mentalization. These theoretical assumptions are consistent with empirical evidence that show a direct effect of mentalization on the degree of adaptive emotional regulation (Schwarzer et al., 2021). Given these connections and peculiarities of these three constructs, it would be appropriate to further investigate the relationship between these variables in PD patients, to obtain a more detailed view of their social cognition abilities.

Using a multidimensional conceptualization of mentalization is also helpful for our classification and treatment of PDs, since PDs could be reconceptualized as a cluster of interacting dimensions rather than in terms of categories. This hypothesis has been previously tested in relation to different psychopathological domains, for example, eating disorders, with promising results (Gagliardini et al., 2020). In this direction, there is consensus among experts that the current diagnoses of PDs lack sufficient validity, reliability, and clinical utility (Hopwood et al., 2018; Hyman, 2010; Morey et al., 2015). There is evidence that PDs are neither categorical nor divisible as discrete types (Haslam et al., 2012; Trull & Durrett, 2005) as postulated in the Diagnostic and Statistical Manual of Mental Disorders–5's Section II (American Psychiatric Association, 2013), and the International Statistical Classification of Diseases and Related Health Problems 10 (ICD‐10; World Health Organization, 1992). Categorical models of PDs are associated with a variety of problems in clinical research and practice, such as extensive heterogeneity within PD diagnoses (Johansen et al., 2004), and high comorbidity of PDs (Clark, 2007; Zimmerman et al., 2005).

These and other critics have moved the authors of the DSM‐5 (APA, 2013) toward the construction of an alternative model for the classification of PDs, which is more dimensionally oriented. In this context they have used mentalization for the operationalization of Criterion A's, the Level of Personality Functioning Scale (LPFS), that was developed based on a review of different measures of personality pathology and personality functioning (Bender et al., 2011). The RFS, a measure of mentalizing (Fonagy et al., 1998), was one of the five instruments examined. The authors of the LPFS have conceptualized Criterion A stating that “[…] the ability to mentalize is important in the assessment of levels of personality functioning” (Bender et al., 2011, p. 338). As outlined in detail below, the LPFS and mentalization share a strong conceptual overlap (Zettl et al., 2019). In line with this, Clarkin et al. (2015) have highlighted that from a clinician's perspective the central issue is to identify the constituent domains of dysfunction related to PDs and the mechanisms underlying these domains that contribute to disruption of internal and relational world.

### 1.1 Aims

In light of the aforementioned considerations the general purpose of this study was to investigate the relationship between the patients' mentalizing problematics, their personality, and the other psychological characteristics.

Furthermore, this study had three specific aims:

‐ identify and describe clusters of individuals characterized by specific patterns of mentalizing imbalance as rated by their clinicians;
‐ analyze the relationship between these clusters and the diagnosis of PD;
‐ analyze the relationship between clusters and other psychological characteristics such as: nonmentalizing modes, emotion dysregulation, and interpersonal reactivity.

## 2 METHOD

### 2.1 Sampling procedure

To collect data, we contacted via e‐mail 2000 psychotherapists from the rosters of the main societies of psychotherapy in Italy and from Centres specialized in the treatment of PDs, and asked for their willingness to participate to the study. Four hundred therapists accepted to participate, for an overall response rate of 20%. We requested that they selected a patient who was at least 18 years old, had no psychotic disorder or psychotic symptoms for at least the last 6 months, had seen the therapist for a minimum of eight sessions, and had a PD diagnosis or a clinically relevant personality problem. To minimize selection biases, we directed the clinicians to consult their calendars to select the last patient they had seen during the previous week who met the study criteria. To minimize rater‐dependent biases, each clinician was allowed to describe only one patient. The final sample is composed of 400 therapeutic dyads. The first part of the study only included clinicians, while the second part of the study included both clinicians and patients, therefore, a part of this sample (*n* = 183) only used clinician‐report measures and did not require the direct participation of patients, while part of the sample (*n* = 217) also fulfilled self‐report measures rated by the patients, after providing their written informed consent to the participation of the study. All PD diagnoses were provided by therapists.

### 2.2 Therapists

The sample is composed of 400 Caucasian therapists, of whom 234 were (58.5%) females and 166 (41.5%) males (mean age = 41.0; SD = 10.67; min. = 22; max. = 68), with a psychodynamic (*n* = 248), cognitive (*n* = 103), and integrative (*n* = 49) theoretical and clinical approach. Therapists had an average of 11.8 years of previous clinical experience as psychotherapists (SD = 10.0; min. = 1; max. = 40). One hundred and sixty‐four (41%) therapists were seeing the selected patient in a private setting, while 156 (39%) in public health settings, 28 in residential structures (7%), 21 in schools (5.3%), and 31 (7.8%) in different contexts.

### 2.3 Patients

The sample is composed of 400 Caucasian patients treated in psychotherapy, of whom 274 were female (68.5%) and 126 males (31.5%) (mean age = 33.0; SD = 11.5; min. = 18; max. = 75). The average length of treatment at the moment of the evaluation was 13.5 months (*SD* = 13.1; min. = 3; max. = 100). Sixty‐three patients (15.8%) had at least one previous hospitalization and 58 (14.5%) two or more. Sixty‐six patients (16.5%) had attempted suicide at least once, 110 were reported with self‐harming behaviors (27.5%), and 197 (49.3%) were, by the time of the assessment, undergoing a pharmacotherapy. Two hundred and forty‐six patients (61.5%) had a PD diagnosis according to the DSM‐5 (APA, 2013), alone or in comorbidity, and 154 (38.5%) patients had clinically relevant problematics in personality (i.e., they fulfilled four out of five criteria for borderline PD or for the obsessive‐compulsive disorder, etc.). The most frequently diagnosed PDs were: avoidant (*n* = 117), borderline (*n* = 87), and obsessive–compulsive (*n* = 57). Three hundred and eighty patients had one or more clinical diagnosis according to the DSM‐5 (APA, 2013). The most common were: depressive disorders (*n* = 187), anxiety disorders (*n* = 177), eating disorders (*n* = 143), and substance use disorders (*n* = 68).

### 2.4 Ethical issues

The clinicians and patients received no remuneration. All of the participants provided written informed consent and all data were treated in an aggregate and anonymous way according to the current law on privacy (GDPR 679/2016). This study project was approved by the “Carlo Bo” University of Urbino Ethical Committee.

### 2.5 Measures

Clinician report measures included the following.

**Clinical Questionnaire**. The clinical questionnaire was constructed ad hoc for clinicians to obtain general information about them, their patients, and the therapies they used. Clinicians provided basic demographic and professional data (e.g., theoretical approach) and additional data on the therapies (e.g., length of treatment). To provide a more comprehensive assessment of patients' problems that may be connected to PDs and/or mentalizing deficits, respondents were also asked to use the items of the Clinical Questionnaire to rate the presence or absence of a list of clinical problems (APA, 2013), such as dissociative symptoms, self‐harming behaviors, and eating disorders.

**PD Checklist**. Following the same procedure adopted in similar studies (e.g., Colli et al., 2014; Gagliardini et al., 2020), we asked clinicians to rate each randomly ordered criterion for each of the DSM–5 PD diagnoses (APA, 2013), as present or absent. This procedure provided both a categorical diagnosis (by applying DSM–5 cutoffs) and a dimensional measure (number of criteria met for each disorder).

**Mentalization Imbalances Scale** (MIS; Gagliardini et al., 2018; Gagliardini, Gatti, & Colli, 2020). The MIS represents a clinician‐report assessment measures of mentalizing imbalances in adult patients, composed of 22 items rated on a 6‐point Likert scale. It includes six subscales: imbalance toward self (four items); imbalance toward others (three items); affective imbalance (four items); cognitive imbalance (five items); automatic imbalance (three items); external imbalance (three items). In the present study, alphas were, respectively, 0.88 (cognitive imbalance), 0.84 (imbalance on the self), 0.84 (affective imbalance), 0.74 (imbalance on the others), 0.74 (automatic imbalance), 0.73 (external imbalance).

**Modes of Mentalization Scale** (MMS; Gagliardini & Colli, 2019; Gagliardini, Gatti, & Colli, 2020). MMS's items were developed in relation to three nonmentalizing modalities (i.e., concrete thought, teleological thought, pseudomentalization), with also items related to good mentalization (Gagliardini & Colli, 2019). However, the exploratory factor analysis conducted during the validation study enlightened the presence of five different scales. Therefore, the MMS is a clinician‐report assessment measure of the modes of mentalization on five different subscales: (1) excessive certainty (six items), indicating an excessive certainty about mental states and other people's inner worlds; (2) concrete thinking (six items), indicating the tendency to interpret reality on the basis of heuristics and prejudices and/or on the basis of physical or invariant constraints, to use common‐sense explanations or clichés to explain emotions, and to adopt bizarre explanations of behaviors; (3) good mentalization (five items), indicating a good capacity to recognize and coherently describe mental states, united with a curious stance toward the same and an awareness that people can experience contrasting feelings and desires; (4) teleological thought (three items), indicating a tendency to rely more on the physical manifestations of mental states (i.e., actions) rather than interpreting the world in terms of beliefs, desires, or thoughts, to focus more on what people do (and not on what they think or feel), and to be more focused on the physical, practical, resolution of a problem rather than on the meanings related to the situation; (5) intrusive pseudomentalization (four items), related to a more malign form of hyper‐ or pseudomentalization, indicating a tendency to intrude on and manipulate other people's life, in which the reflections of one's inner world do not seem to be genuine. In the present study, alphas were, respectively, 0.90 (excessive certainty), 0.86 (good mentalization), 0.83 (concrete comprehension), 0.77 (teleological thought), and 0.73 (intrusive pseudomentalization).

Self‐report measures included the following.

**Reflective Functioning Questionnaire** (RFQ; Fonagy et al., 2016; Morandotti et al., 2018), for the assessment of mentalization from the patient's perspective. It assesses mentalization or reflective function by asking the patient to answer to 8 items on a 7‐point Likert scale. Scores are then recoded and collapse into two different subscales: RFQ_certainty (RFQ_c, six items) which reflects an excessive certainty about mental states; and RFQ_uncertainty (RFQ_u, six items) which reflects an excessive uncertainty about self and others' mental states. In this study alphas were, respectively, 0.69 (RFQ_u) and 0.72 (RFQ_c).

**Interpersonal Reactivity Index** (IRI; Albiero et al., 2006; Davis, 1980), for the assessment of empathic responsiveness, is composed of 28 items rated by patients on a 5‐point Likert scale. It is composed of four subscales, with seven items each: (1) phantasy, that assess the tendency to transpose oneself into an imaginary situation; (2) empathic concern, that assess the tendency to feel sympathy and compassion toward others; (3) perspective taking, that assess the tendency to assume the point of view of others; (4) personal distress, that assess the tendency to feel distress in reaction to the distress of the others. In this study, alphas were, respectively, 0.73 (phantasy), 0.64 (empathic concern), 0.81 (perspective taking), and 0.72 (personal distress).

**Difficulties in Emotion Regulation Strategies** (Gratz & Roemer, 2004; Sighinolfi et al., 2010), composed of six subscales for the assessment of emotional regulation: (1) lack of acceptance of the emotional response (“nonacceptance”); (2) difficulty in distracting from emotions and to engage in goal‐oriented behaviors (“goals”); (3) limited response to emotional regulation strategies (“strategies”); (4) lack of control when experiencing intense emotions (“impulse”); (5) difficulties in recognizing emotions (“clarity”); (6) limited awareness of emotion (“awareness”). In this study, we adopted the Italian version of the scale, which has been validated by Sighinolfi et al. (2010). In this study, alphas were, respectively, 0.89 (nonacceptance), 0.85 (goals), 0.89 (strategies), 0.88 (impulse), 0.83 (clarity), and 0.81 (awareness).

### 2.6 Data analysis

Preliminary analyses were conducted to verify factor structure of the MIS, recently developed and validated (Gagliardini et al., 2018; Gagliardini, Gatti, & Colli, 2020) that underpin the present work. Model fit was based on the following goodness of fit indices (e.g., Marsh et al., 2005; Schermelleh‐Engel et al., 2003): the Tucker‐Lewis index (TLI; comparative fit index [CFI] ≥ 0.97 indicates good fit; ≥0.95 indicates acceptable fit), the root mean square error of approximation (RMSEA; RMSEA ≤ 0.05 indicates good fit; ≤0.08 indicates acceptable fit), and the 90% confidence interval of the RMSEA.

Latent Profile Analysis (LPA; Lanza et al., 2003) was used to identify latent variables that represent classes (or clusters) of individuals who share similar MIS profiles. LPA identifies classes of people via maximum likelihood estimation (Little & Rubin, 1987) using continuous dependent variable. The probability that an individual was properly classified, which enables each person to be categorized into the best‐fitting class, is estimated simultaneously with the overall model (Hill et al., 2006). Models are estimated with classes added iteratively to determine which model is the best fit to the data. For this study, LPA was conducted using MPlus 6.1 (Muthén & Muthén, 2006). To determine the optimal number of classes for the sample, each model was evaluated using the Lo‐Mendell‐Rubin Adjusted Likelihood Ratio Test (LMRT; Lo et al., 2001), the Bootstrapped Likelihood Ratio Test (BLRT; Arminger et al., 1999; McLachlan & Peel, 2000), Akaike information criteria (AIC; Akaike, 1974), and sample size‐adjusted Bayesian information criteria (sBIC; Schwarz, 1978). The LMRT and the BLRT compare the fit of a target model (e.g., 2 class model) to a comparison model which specifies one less class (e.g., 1 class model). The *p*‐value generated for the LMRT and BLRT indicates whether the solution with more (*p* < 0.05) or less classes (*p* > 0.05) fits better. The AIC and sBIC are descriptive fit indices in which smaller values indicate better model fit. Moreover, each model was evaluated on their interpretability to verify if the classes truly represented different categories, rather than being a bias resulting from distribution (Muthén, 2006). LPA was conducted on the overall sample considering it as “sufficiently large” to ensure good parameter recovery with few indicators (Wurpts & Geiser, 2014). Moreover, to handle the possible presence of small classes (those that contain less than 5% of the sample) was also considered when determining the optimal number of classes to avoid too many classes/profiles (Hipp & Bauer, 2006). General Linear Model (analysis of variance [ANOVA] and two‐way Multivariate analysis of variance [MANOVA]) were used to analyze differences among the factors (i.e., clinical characteristics and resulting profiles) in emotional regulation, empathic responsiveness, adequate mentalization and nonmentalization modes.

## 3 RESULTS

### 3.1 Preliminary Confirmatory Factor Analysis

The six‐factor model of the Mentalization Imbalances Scale (MIS) was preliminary tested. The model included the six covariances reported in the first validation of the MIS (Gagliardini et al., 2018). Fit indices *χ* ^2^_(188)_ = 531.750 (*p* < 0.05), CFI = 0.921, TLI = 0.908, RMSEA = 0.068 (90%CI = 0.061‐0.074, and SRMR = 0.078 loadings of the MIS were all significant and above 0.53. These values were similar to those previously obtained and confirm that the present factor structure is sufficient, but not excellent.

### 3.2 LPA

To identify profiles of patients with similar mentalization capacity, an LPA was performed on the entire sample using six indicator variables (i.e., the six mentalization imbalances assessed through the MIS subscales: affective, cognitive, automatic and external imbalances, imbalance toward self and toward others; see Measures section); the model fit indices are shown in Table 1. Regarding MIS, LPA showed that the LMRT and BLRT indicated that the 2‐class, 3‐class, and 4‐class solution fit better than the previous (*x* − 1) class solution, respectively, the one‐class, the 2‐class, and the 3‐class solutions. The 4‐class solution resulted superior to the 3‐class solution due to a significant LMRT value (*p* = 0.04) and lower AIC and BIC values. Although the 5‐class solution revealed slightly lower AIC value and a statistically significant BLRT value (*p* = 0.00), the LMRT indicated that it was not statistically different from the 4‐class solution (*p* = 0.18) and BIC value resulted slightly higher than in the 4‐class solution. Therefore, the 4‐class solution was considered the best fit to the data.

**Table 1:** Model fit for each LPA model (*N* = 400)

Nr of classes	LMRT	BLRT	AIC	BIC
MIS				
1	‐	‐	7661.91	7709.81
2	366.86 (0.00)	376.00 (0.00)	7300.31	7376.15
3	165.98 (0.00)	169.94 (0.00)	7144.37	7248.15
4	89.96 (0.04)	92.10 (0.00)	7066.26	7197.98
5	38.98 (0.18)	39.91 (0.00)	7040.35	7200.01

Abbreviations: AIC, Akaike information criteria; BIC, Bayesian information criteria; BLRT, Bootstrapped Likelihood Ratio Test; LMRT, Lo‐Mendell‐Rubin Adjusted Likelihood Ratio Test; MIS, Mentalization Imbalances Scale; MMS, Modes of Mentalization Scale.

**Figure 1:**
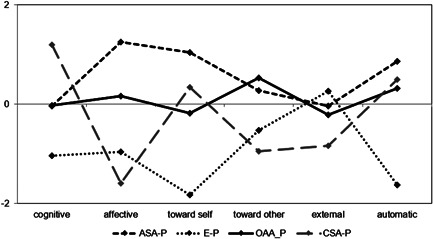
Graphic representation of mean‐centered parameter estimates for MIS Profiles (*N* = 400). ASA‐P, Affective Self Automatic Profile; CSA‐P, Cognitive Self Automatic Profile; E‐P, External Profile; MIS, Mentalization Imbalances Scale; OAA‐P, Others Automatic Affective Profile.

The mean centered estimates with medium to large effect were used to interpret each profile, see Table 2 and Figure 1. Regarding the MIS, the 4‐class solution identified profiles characterized by different combinations of imbalances. The first class was composed of 31.7% of the sample (*n* = 127) and represented individuals with the highest scores in affective, toward‐self and automatic scales of MIS (we will refer to this cluster as Affective‐Self‐Automatic Profile [ASA‐Profile]). The second class was composed of 18.3% of the sample (*n* = 73) and represented individuals with a peak in the external imbalance (named E‐Profile). The third class, composed of 32.5% of the sample (*n* = 130), was characterized by individuals who reported the highest scores in the imbalance toward others scale and relative high scores in the automatic and affective MIS scales (Other‐Automatic‐Affective Profile [OAA‐Profile]). Finally, the last class, composed of 17.5% of the sample (*n* = 70), represented individuals with high scores in cognitive, toward‐self, and automatic scales of MIS (Cognitive‐Self‐Automatic Profile [CSA‐Profile]).

**Table 2:** Mean‐centered parameter estimates for MIS profiles (*N* = 400)

	Overall sample	ASA‐P (*n* = 127)	E‐P (*n* = 73)	OAA‐P (*n* = 130)		CSA‐P (*n* = 70)
Cognitive imbalance	2.44	−0.04	−1.04	−0.02		1.20
Affective imbalance	2.69	0.26	−0.96	0.17		−1.59
Imbalance toward self	2.74	1.04	−1.83	−0.18		0.34
Imbalance toward others	2.12	0.28	−0.52	0.53		−0.95
External imbalance	2.16	−0.03	0.27	−0.21		−0.84
Automatic imbalance	2.43	0.87	−1.62	0.32		0.50

Abbreviations: ASA‐P, Affective Self Automatic Profile; CSA‐P, Cognitive Self Automatic Profile; E‐P, External Profile; MIS, Mentalization Imbalances Scale; OAA‐P, Others Automatic Affective Profile.

### 3.3 Explore characteristics between subjects with and without PDs

To analyze the relationship between the classes identified and the diagnosis of PD, the distribution of the four classes was compared between subjects with and without PDs. Results showed statistically significant differences in the distributions of the two subsamples across the four classes (*χ* ^2^ = 48.23, *p* = 0.000). Concerning subjects with PDs, 42.7% are in the ASA‐Profile, 31.3% in the OAA‐Profile, 15.9% in the CSA‐Profile, and 10.2% in the E‐Profile. Regarding subjects without a diagnosis of PDs, 34.4% in the OAA‐Profile, 31.2% in the E‐Profile, 20.1% in the CSA‐Profile, and 14.3% are in the ASA‐Profile. ASA‐profiles (*Δ* = +28.4% in PD sample) and E‐profiles (*Δ* = +21.0% in non‐PD sample) showed the greater differences between the two subgroups, while the percentage of the OAA and CSA were similar (*Δ* = 3/4%).

Two‐way MANOVA was performed to analyze whether mentalization imbalances differ with regard to presence of PDs as main effect and in interaction with classes of mentalization. To handle the violations of equality of variances and multivariate normality, we set a more conservative *p*‐values (0.01 instead of 0.05) for significance testing and interpreted Pillai's trace instead of Wilks's *λ*, a more robust statistic for comparison, respectively (Tabachnick & Fidell, 2007). The results of the two‐way MANOVA indicated neither interaction effect (Pillai's trace = 0.07, *p* = 0.11, *η* ^2^ = 0.02) nor effect of PD presence (Pillai's trace = 0.04, *p* = 0.02, *η* ^2^ = 0.04), while, as expected, class of mentalization showed a significant effect (Pillai's trace = 0.1.39, *p* < 0.01, *η* ^2^ = 0.46). Taken together, these results seem to indicate that the presence of PD does not affect specifically the elevation of mentalization imbalances of the classes of subjects, but rather that it is associated with the predominance of a certain type of mentalization profile characterized by strong affective imbalance and by an imbalance on the self.

### 3.4 Personality pathology and mentalization profiles

Distributions of PDs among the identified MIS profiles showed a picture of significant associations between these two classifications (Table 3; *p*_s_ < 0.01) with the exception of Histrionic, Avoidant, and Obsessive disorders. The prevalence of MIS profiles within the diagnostic labels varied across disorders showing higher percentage of ASA‐P profile in Paranoid, Schizotypal, Antisocial, Narcissistic, and Dependent disorders, higher percentage of CSAP profile in Schizoid and Obsessive, and higher percentage of OOA profile in Avoidant and Obsessive disorders.

**Table 3:** Distribution of PDs among the MIS profiles (*N* = 400)

	ASA‐P (%)	E‐P (%)	OAA‐P (%)	CSA‐P (%)	*χ* ^2^_(3)_
Paranoid	0.70	0.00	0.16	0.14	30.19**
Schizoid	0.25	0.08	0.25	0.42	22.87**
Schizotypal	0.59	0.00	0.18	0.23	11.56**
Antisocial	0.76	0.05	0.14	0.05	36.39**
Borderline	0.68	0.03	0.28	0.01	76.71**
Histrionic	0.52	0.07	0.33	0.07	7.24
Narcissistic	0.63	0.07	0.19	0.11	13.17**
Dependent	0.55	0.04	0.38	0.04	24.26**
Avoidant	0.33	0.11	0.39	0.18	6.47
Obsessive–compulsive	0.23	0.21	0.28	0.28	6.60
Presence of PD	0.43	0.01	0.31	0.16	48.23**

Abbreviations: MIS, Mentalization Imbalances Scale; PD, personality disorder.

**p < 0.01.

To pursue the third aim of the study we analyzed the relationship between the four clusters identified and the patients' psychological characteristics rated by their clinician (MMS) or by themselves (RFQ, DERS, and IRI).

### 3.5 Nonmentalization modes

To examine profile differences in nonmentalization modes (MIS), we carried out a one‐way MANOVA (see Table 4). The model was significant as assessed with Wilks *λ* of 0.456 (*p* = 0.000; *η_p_* ^2^ = 0.230). Test of between subjects effects indicated that nonmentalization differed significantly across all profiles for all modes: excessive certainty (*F*_(3, 396)_ = 48.73; *p* = 0.000; *η_p_* ^2^ = 0.270), concrete think (*F*_(3, 396)_ = 70.215; *p* = 0.000; *η_p_* ^2^ = 0.347), teleological (*F*_(3, 396)_ = 63.851; *p* = 0.000; *η_p_* ^2^ = 0.326), and pseudomentalization (*F*_(3, 396)_ = 45.607; *p* = 0.000; *η_p_* ^2^ = 0.374). Similarly, capacity of good mentalization also differed significantly across the different profiles (*F*_(3, 396)_ = 78.768; *p* = 0.000; *η_p_* ^2^ = 0.374).

**Table 4:** Mean, standard deviations, and MANOVA for significant effect of MIS profiles on MMS (*N* = 400)

MMS	ASA‐Profile		E‐Profile		OAA‐Profile		CSA‐Profile		Between subjects		
	*M*	SD	*M*	SD	*M*	SD	*M*	SD	*F*_(3, 396)_	*p*	*η_p_* ^2^
Excessive certainty	2.94	1.01	1.14	0.83	2.26	1.05	2.43	1.14	48.730	0.000	0.270
Concrete think	2.75	0.91	0.93	0.61	1.94	0.85	2.25	1.02	70.215	0.000	0.347
Good mentalization	2.14	0.90	3.83	0.84	2.93	0.67	2.14	0.94	78.768	0.000	0.374
Teleological	3.57	0.91	1.56	0.99	2.55	1.05	2.92	1.14	63.851	0.000	0.326
Pseudomentalization	2.41	0.96	0.75	0.87	1.64	1.03	2.02	1.11	45.607	0.000	0.257

*Note*: Multivariate analysis of variance (MANOVA) Wilkes' *λ* = 0.456, *p* = 0.000. ASA, *n* = 127; E, *n* = 73; OAA, *n* = 130; CSA, *n* = 70.

Abbreviations: ASA, Affective‐toward Self‐Automatic imbalance; CSA, Cognitive‐toward Self‐Automatic imbalance; E, External imbalance; MIS, Mentalization Imbalances Scale; MMS, Modes of Mentalization Scale; OAA,  Toward Other‐Automatic‐Affective imbalance.

Post hoc analysis (Tukey's multiple comparison test) revealed that subjects high in affective imbalance (ASA‐Profile) reported higher levels of excessive certainty, concrete think, teleological and pseudomentalization modes than those in the OAA‐Profile (*p*_s_ = 0.000), in the CSA‐Profile (p_s_ = 0.000) and in the E‐Profile (*p* = 0.004, *p* = 0.001, *p* = 0.000, *p* = 0.039, respectively). Similarly, this latter cluster, showed lower levels of excessive certainty, concrete think, teleological and pseudomentalization modes than subjects in the OAA‐Profile (*p*_s_ = 0.000) and in the CSA‐Profile (*p*_s_ = 0.000). Finally, subjects in the OAA‐Profile and in the CSA‐Profile differed from each other only in pseudomentalization mode, higher in the last cluster (*p* = 0.049).

Capacity of good mentalization was higher in individuals belonging to the E‐Profile, compared to those belonging to the OAA‐Profile (*p* = 0.000), CSA‐Profile (*p* = 0.000) and ASA‐Profile (*p* = 0.000). This latter cluster showed lower levels of good mentalization also compared to OAA‐Profile (*p* = 0.000), which, however, showed higher levels than CSA‐Profile (*p* = 0.000).

### 3.6 Differences across profiles on self‐report measures

Regarding the second aim of our study, the relationship between clinician's and patient's perception of the patient's clinical characteristics was explored by examining differences among the profiles in mean scores of the self‐report measures (RFQ, DERS, and IRI) (see Table 5). The one‐way MANOVA was conducted on a subsample of 217 participants for which the three aforementioned self‐report were available.

**Table 5:** Mean, standard deviations, and MANOVA for significant effect of MIS profiles on reflective functioning, difficulties in emotion regulation, interpersonal reactivity (*N* = 217)

Measure	ASA‐Profile		E‐Profile		OAA‐Profile		CSA‐Profile		Between subjects		
	*M*	SD	*M*	SD	*M*	SD	*M*	SD	*F*_(3, 217)_	p	*η_p_* ^2^
RFQ‐uncertainty	1.21	0.75	0.78	0.63	0.96	0.60	0.93	0.68	5.26	0.00	0.07
RFQ‐certainty	0.64	0.71	0.87	0.71	0.87	0.69	1.17	0.80	3.66	0.01	0.5
DERS‐nonacceptance	3.21	1.06	2.88	1.11	3.05	1.10	2.52	1.15	2.85	0.04	0.04
DERS‐strategies	3.19	1.01	2.84	0.88	2.92	0.86	2.46	0.76	4.46	0.00	0.06
DERS‐impulsivity	3.18	1.04	2.56	0.99	2.77	1.01	2.67	1.03	12.69	0.00	0.15
IRI‐empathic concerns	3.73	0.61	3.77	0.66	3.86	0.57	3.43	0.70	5.26	0.00	0.07

*Note*: Multivariate analysis of variance (MANOVA) Wilkes' *λ* = 0.90, *p* = 0.002; 0.79, *p* = 0.000; 0.91, *p* < 0.05. ASA, *n* = 48; E, *n* = 61; OAA, *n* = 75; CSA, *n* = 33.

Abbreviations: ASA, Affective‐toward Self‐Automatic imbalance; CSA, Cognitive‐toward Self‐Automatic imbalance; DERS, Difficulties in Emotion Regulation Scale; E, External imbalance; IRI, Interpersonal Reactivity Index; OAA,  Toward Other‐Automatic‐Affective imbalance; RFQ, Reflective Functioning Questionnaire.

The models were significant as assessed with Wilks *λ* of 0.90 for RFQ (*p* = 0.002; *η_p_* ^2^ = 0.05), 0.79 for DERS (*p* = 0.000; *η_p_* ^2^ = 0.08), and 0.91 for IRI (*p* = 0.048; η_p_^2^ = 0.03). Test of between subjects effects indicated that reflective functioning mean scores differed significantly across all profiles for both certainty (*F*_(3, 217)_ = 3.66, *p* < 0.05, *η* ^2^ = 0.05) and uncertainty (*F*_(3, 217)_ = 5.26, *p* < 0.01, *η_p_* ^2^ = 0.07) scores. Post hoc analysis (Tukey's multiple comparison test) revealed that subjects high in affective imbalance (ASA‐Profile) reported higher levels of uncertainty than did subjects high in external (E‐Profile) (*p* = 0.005) and in cognitive imbalances (CSA‐Profile) (*p* = 0.004), whereas in this latter cluster subjects had higher level in certainty than those in the ASA‐profile (*p* = 0.006). Test of between subjects effects indicated that the profiles differed on DERS nonacceptance (*F*_(3, 217)_ = 2.85, *p* < 0.05, *η_p_* ^2^ = 0.04), strategies (*F*_(3, 217)_ = 4.46, *p* < 0.01, *η_p_* ^2^ = 0.06) and impulse (*F*_(3, 217)_ = 12.69, *p* < 0.01, *η_p_* ^2^ = 0.15). Follow‐up Tukey's significant difference comparisons for DERS showed that subjects in ASA‐Profile had higher nonacceptance, lack of strategies (or self‐confidence) and impulsivity than subjects in CSA‐Profile (*p* = 0.030, *p* = 0.002, and *p* = 0.000, respectively). Moreover, the latter cluster showed lower impulsivity also with regard to the other two profiles (E‐P and OAA‐P) (*p* = 0.006 and *p* = 0.000, respectively). Finally, only the subscale empathic concern of the IRI showed significant mean difference among the four profiles (*F*_(3, 217)_ = 3.55, *p* < 0.05, *η_p_* ^2^ = 0.05), with greater concerns in other imbalance cluster (OAA‐P) than in cognitive profile (CSA‐P) (*p* = 0.008).

## 4 DISCUSSION

The first aim of this study was to investigate the presence of mentalization profiles in patients with personality pathology based on the perception of their clinician during the psychotherapy. LPA results in relation to the MIS (which assesses mentalizing imbalances) indicated the presence of four clusters: (1) Affective‐Self‐Automatic Profile (ASA‐P), (2) External Profile (E‐P), (3) Other‐Automatic‐Affective Profile (OAA‐P), (4) Cognitive‐Self‐Automatic Profile (CSA‐P). Supporting Information: Appendix A shows the main characteristics of PDs and mentalizing problems for each cluster. The first (ASA‐P) and the third (OAA‐P) cluster seem to share the imbalance on the affective polarity as well as the tendency to rely more on automatic mentalization process. However, subjects in the ASA‐P in doing so are more focused on the self, while those in the OAA‐P seem to be more focused on the others. Our results also indicated the presence of a difference in the affective imbalance between these two profiles (higher in ASA than in OAA), but we do not know whether the difference in the object of mentalization (self or other) may indicate a different solution at the attempt to regulate the affective experience or whether the different imbalance on the self or on the others may cause problematics in automatic and affective mentalization. The fourth cluster (CSA‐P) seems to indicate patients with specular and opposite problematics, in which the focus on the cognitive dimension is predominant, whereas the third one (E‐P) is represented by patients characterized by an imbalance on the external dimension. This cluster seems to include patients who are hypervigilant toward others. Compared to the ASA‐P patients, E‐patients do not seem to show problematics in the affective and automatic dimensions of mentalization and seem to be more focused on the external cues of others than on their own self. The mentalizing profiles differ significantly in relation to clinical variables assessed throughout self‐report measures, that is, emotion regulation, certainty/uncertainty about mental states, and interpersonal sensibility. In particular, the excessive uncertainty about mental states seems to characterize the ASA cluster, while excessive certainty seems to be predominant in the CSA cluster.

In relation to the quality of mentalization (i.e., the presence of prementalizing modalities of thought), we found that subjects high in affective imbalance (ASA‐Profile) reported higher levels of excessive certainty, concrete think, teleological, and pseudomentalization modes than those in the OAA‐Profile, in the CSA‐Profile, and in the E‐Profile. Similarly, this latter cluster, shows lower levels of excessive certainty, concrete think, teleological, and pseudomentalization modes than subjects in the OAA‐Profile and in the CSA‐Profile. Finally, subjects in the OAA‐Profile and in the CSA‐Profile differ from each other only in pseudomentalization mode, higher in the last cluster. These results may indicate that the ASA‐P represents the profile with more significant problematics in mentalization, compared to other profiles. The CSA profile seems to describe detached and introjective patients, who are more focused on the self and rely more on abstract reasoning than on emotions, compared to ASA‐P patients who seem to be characterized by an anaclitic style (Blatt, 2004; Luyten & Blatt, 2013). This result is also confirmed by the high levels of pseudomentalization in CSA‐P patients. These two profiles differ in terms of the predominance of the imbalances on the cognitive or affective dimensions, and seem to be characterized by a fair higher presence of problematics in mentalization. The E‐profile shows some peculiar characteristics that differentiate it from the other three profiles. It can be assumed that this profile seems to involve patients with specific capacities of mentalization, it is indeed the only profile that does not have high scores on the automatic scale, suggesting that individuals in this cluster have a relative greater tendency to be aware and controlled, or in other words, these individuals may be hypervigilant. Another hypothesis, not necessarily in contrast to the first, is that this profile includes subjects with good mentalizing qualities and a better mental health status. In partial support of this interpretation, we can note that the percentage of patients in E‐profile is three times higher among patients without PD (30%) than among those with PD (10%). Finally, it should be noted that the self‐reports results are consistent with these hypotheses, showing that patients self‐represent themselves as less impulsive (DERS) and with a greater degree of certainty (RFQ) in comparison with what patients of the other clusters report.

The second aim of our study was to see how these profiles interact with personality pathology. First of all, our results highlight that the association between PD diagnosis and mentalizing profiles does not seem to uniquely reflect the proposed differentiation of the DSM‐5 clusters: for example, the most frequent diagnoses in the ASA profile are paranoid and antisocial, which belong to different DSM‐5 clusters. These data seem to point toward the poor validity of the DSM‐5 clusterization of PDs, which has been addressed by previous work (Widiger & Trull, 2007).

It is also interesting to see how different PDs are characterized by very specific patterns of imbalances in mentalization. Patients with a paranoid or antisocial PD diagnosis were collocated more in the ASA profile, and this partially confirms all those theoretical and empirical contributions on the specific relationships between different PDs and mentalizing problems (Bateman & Fonagy, 2016; Semerari et al., 2014). Other PDs, such as avoidant and dependent PDs, were instead less specifically related on one single cluster and tended to be represented in more than one cluster. This may be interpreted both as an indicator of the presence of different subtypes of the same PD in relation to mentalization or as the signal of the presence of different moments related to different modalities of functioning in patients with the same diagnosis. High percentage of patients with Narcissistic or Borderline diagnosis fall in ASA profile, and while the self‐focus of these patients is not surprising, the affective imbalance that was found in narcissistic patients could be linked to the attempt to inflate self‐worth (to avoid unmentalized feelings of inadequacy) through the massive use of nonmentalizing modes (e.g., pseudomentalization) (Lecours et al., 2013), or alternatively as the expression of the problematics in affective mentalization described by different authors (Drozek & Unruh, 2020). In a similar way, it is inconsistent with the results of the literature (Dimaggio et al., 2007) the fact that the highest percentage of avoidant patients falls in the mentalization profile characterized by an imbalance toward others.

Our results also indicated that patients with PDs, compared to non‐PD patients, were characterized by qualitative differences in the prevalence of specific MIS profiles. Taken together, these results seem to indicate that the presence of PDs does not affect specifically the elevation of mentalization imbalances of the classes of subjects, but rather that it is associated with the predominance of a certain type of mentalization profile characterized by strong affective imbalance and by an imbalance on the self. These results confirm that PDs are related to specific differences in the quality of mentalization, which can be addressed with specific treatment strategies, such as in mentalization‐based treatment or treatment focused on the impairments of mentalization.

## 5 CLINICAL IMPLICATION

These data seem to be helpful both from a diagnostic and from a clinical perspective. The assessment of mentalization helps us at tailoring the intervention more carefully: We may interact with patients who have the same diagnosis but are on different mentalizing levels or, on the contrary, with patients with different diagnoses but with similar problems in mentalization. This study also provides interesting cues on how imbalances are interpreted by the clinician's perspective, for example, the results relating mentalization imbalances of narcissistic and avoidant patients might suggest that clinicians focused and rated the struggle that these patients undertake to manage their specific deficits.

## 6 LIMITATIONS OF THE STUDY

The main limitation of this study is related to the fact that PD diagnosis was gained from clinicians, and not throughout self‐reports fulfilled by the patients. Research has previously shown that clinicians prefer to rely on global impressions based on their unstructured interviews and subjective experience of interactions with the patient, rather than systematically assessing diagnostic criteria (First et al., 2004; Samuel & Bucher, 2017). This problematic is partially mitigated by the fact that clinicians have signed as present or absent each DSM‐5 criterion for PDs randomly ordered (we did not rely only on their evaluation of the presence or absence of a diagnosis.

A different limitation is related to the fact that nearly half of the sample was composed only by clinicians and did not come with data on self‐report measures: the risk of a circularity of the answers provided by a single informant (clinician) is present. Although the results that emerged from the analyses that we conducted on the self‐reports were coherent and have substantially confirmed our results, we cannot consider this limitation as fully mitigated. Finally, it must be emphasized that in interpreting the results we have considered above all similarities among individuals within the same cluster, but this could represent an underestimation of the differences between subjects in the same cluster.

Lastly, a limitation of the study is the low alpha values for some self‐report scales (RFQ_u, RFQ_c, IRI empathic concern). Despite these limitations, however, our study suggests and partially confirms the idea that different PDs can be reconceptualized on the basis of mentalizing problematics. For the future, it should be necessary to test the predictive validity and the response to treatment of these mentalizing profiles, to construct and validate therapies which are more focused on the psychopathological domains concerned and rather than on diagnoses alone.

## CONFLICT OF INTEREST

The authors declare no conflict of interest.

### 1 PEER REVIEW

The peer review history for this article is available at https://publons.com/publon/10.1002/jclp.23430

## Supporting information

Supporting information.

## Data Availability

The data that support the findings of this study are available from the corresponding author upon reasonable request.
