# Supplementary material for: Personality and mentalization: A latent profile analysis of mentalizing problematics in adult patients
Source: J Clin Psychol. 2022 Aug 17;79(2):514–30. doi: 10.1002/jclp.23430 (PMC10087971; doi:10.1002/jclp.23430)
Supplement: Supplementary file 1 — Supporting information. [file JCLP-79-514-s001.docx]

| Appendix A  *Mentalization profiles, % of PDs for each cluster and problematics in the imbalances of mentalization (MIS) and non-mentalizing modes (MMS) for each cluster* | | | | | | |
| --- | --- | --- | --- | --- | --- | --- |
| **Cluster** | **% of the sample** | **% of the sample with PDs** | **% of the sample without PDs** | **Principal PDs** | **Imbalances of mentalization (MIS)** | **Non-mentalization modes (MMS)** |
| **ASA-P** | 31.7 | 42.7 | 14.3 | - Paranoid - Schizotypal - Antisocial - Narcissistic - Dependent | - Affective - Toward self - Automatic | - High levels of excessive certainty, concrete think, teleological and pseudomentalization modes (higher than other three clusters) - High levels of good mentalization (lower than E-P and OAA-P but equivalent to CSA-P) |
| **E-P** | 18.3 | 10.2 | 31.2? |  | - External | - Low levels of excessive certainty, concrete think, teleological and pseudomentalization modes (lower than other three clusters) - High levels of good mentalization (higher than other three clusters) |
| **OAA-P** | 32.5 | 31.3 | 34.4 | - Avoidant - Obsessive | - Toward others - Automatic - Affective | - Low levels of excessive certainty, concrete think, teleological and pseudomentalization modes (lower than ASA-P and CSA-P but higher than E-P) - High levels of good mentalization (higher than ASA-P and CSA-P but lower than E-P) |
| **CSA-P** | 17.5 | 15.9 | 20.1 | - Schizoid - Obsessive | - Cognitive - Toward self - Automatic | - Low levels of excessive certainty, concrete think, teleological and pseudomentalization modes (lower than ASA-P but higher than E-P and OOA-P) - Low levels of good mentalization (lower than E-P and OAA-P but equivalent to ASA-P) |

Note: PD = Personality Disorder; MIS = Mentalization Imbalances Scale; MMS = Modes of Mentalization Scale; ASA-P = Affective Self Automatic Profile, E-P = External Profile; OAA-P = Others Automatic Affective Profile; CSA-P = Cognitive Self Automatic Profile
